# Elements of Latin

**Published:** 1898-06

**Authors:** 


### BOOK NOTICES.

ELEMENTS OF LATIN. For Students of Medicine and Pharmacy. By George D. Crothers, A. M., M. D., Teacher of Latin and Greek in the St. Joseph (Mo.) High School; formerly Professor of Latin and Greek in the University of Omaha; and Hiram H. Bice, A. M., Instructor in Latin and Greek in the Boys' High School of New York City. $5\frac{1}{4}$x$7\frac{1}{2}$ inches. Pages xii-242. Flexible Cloth, $1.25 net. The F. A. Davis Co., Publishers, 1914-16 Cherry Street, Philadelphia; 117 W. Forty-second Street, New York City; 9 Lakeside Building, 218-220 S. Clark Street, Chicago, Ill.

This excellent little book is especially designed for students whose early training in Latin has been neglected.

It is arranged so as to give in the " briefest possible compass those principles of

Latin etymology and construction which are essential to an intelligent use of the terminology of pharmacy and medicine." Of course those who take up this little book need not expect to find in it a preparation of the student for the enjoyment of the literature of the Latin language. It is intended solely to prepare the student for the understanding of and making familiar with the intricate names found in anatomy, botany, and materia medica.

The author, in his preface, thinks that half the difficulty found in anatomy is due to the intricacy of the names given to the different parts. If these be understood by a careful study of the exercises in this book, the mastery of the science of anatomy is made more certain.

In this book there are exercises on special subjects. The author calls attention to several of these: as the eye, the ear, obstetrics, and surgery.

A chapter on prescription writing is given, which is very instructive. There is a list of anatomical proper names with their origin.

We cordially recommend this book to all earnest students who desire to be well educated in the science and art of medicine.

